# Association between triglyceride-glucose index and risk of chronic kidney disease: a meta-analysis

**DOI:** 10.1080/0886022X.2025.2572356

**Published:** 2025-10-29

**Authors:** Yu Zhang, Rongrong Yuan, Dandan Li

**Affiliations:** a Department of Nephrology, Huaihe Hospital of Henan University, Kaifeng, China; b Department of Endocrinology, Huaihe Hospital of Henan University, Kaifeng, China

**Keywords:** Triglyceride-glucose index, chronic kidney disease, incidence, insulin resistance, meta-analysis

## Abstract

The triglyceride-glucose (TyG) index, a surrogate marker for insulin resistance, has been increasingly investigated for its predictive value in chronic kidney disease (CKD). However, many existing studies are cross-sectional, and the longitudinal evidence remains inconclusive. This meta-analysis aimed to evaluate the association between baseline TyG index and the incidence of CKD in adults. A systematic search of PubMed, Embase, and Web of Science was conducted through March 2025. Longitudinal cohort studies assessing the relationship between TyG index and CKD risk in adults were included. Random-effects models were used to calculate pooled risk ratios (RRs) with 95% confidence intervals (CIs). Thirteen datasets from 12 cohort studies (*n* = 58,838) were included. A high TyG index at baseline was associated with a significantly increased risk of CKD during follow-up (RR: 1.44, 95% CI: 1.33–1.56) with mild heterogeneity (I^2^ = 7%). This association remained robust in sensitivity analyses and in prospective studies alone (RR: 1.55, 95% CI: 1.37–1.75). Subgroup analyses showed consistent associations across different populations, age groups, BMI categories, and TyG cutoff definitions. Notably, a stronger association was observed in studies with follow-up durations < 4 years compared to those with longer follow-up (RR: 1.61 vs. 1.29; *p* for subgroup difference = 0.004), suggesting that the predictive value of TyG index is more evident in shorter-term risk assessment. In conclusions, elevated TyG index is independently associated with an increased risk of incident CKD in adults, particularly within shorter follow-up durations, highlighting its potential utility in early risk stratification.

## Introduction

Chronic kidney disease (CKD) has become a major global public health concern, affecting approximately 13% of the adult population worldwide [1,2]. The burden of CKD continues to rise due to aging populations and the increasing prevalence of hypertension, diabetes, and obesity [3]. CKD is associated with elevated risks of cardiovascular disease, kidney failure, and premature mortality, contributing to substantial health care costs and diminished quality of life [4,5]. Early identification and management of modifiable risk factors are essential to slow disease progression and reducing the global burden [5]. Although traditional risk factors such as hypertension, diabetes, and hyperlipidemia are well established [5], recent studies have highlighted the role of metabolic disturbances—particularly insulin resistance—in the pathogenesis of CKD [6].

Insulin resistance, a hallmark of metabolic syndrome, has been implicated in the development and progression of CKD through several interrelated mechanisms, including glomerular hyperfiltration, inflammation, oxidative stress, endothelial dysfunction, and activation of the renin–angiotensin–aldosterone system [6–8]. Given the complexity of direct measurement of insulin resistance, surrogate markers are increasingly used in both research and clinical practice [9]. The triglyceride-glucose (TyG) index, calculated as ln [fasting triglycerides (mg/dL) × fasting glucose (mg/dL)/2], has emerged as a simple, reliable, and cost-effective marker of insulin resistance [10]. The TyG index shows strong correlation with the hyperinsulinemic-euglycemic clamp technique, the gold standard for IR assessment, and has been proposed as a valuable tool for early detection of metabolic dysfunction [11,12]. Its clinical utility has been explored in various conditions, including cardiovascular disease [13], nonalcoholic fatty liver disease [14], and more recently, CKD [15]. Despite growing interest, the relationship between TyG index and CKD remains inadequately defined. Most existing studies on this topic are cross-sectional in design, limiting the ability to establish a temporal or causal link [15]. While some longitudinal studies have suggested a potential association between elevated TyG index and incident CKD [16–25], the results are not entirely consistent [26,27]. Therefore, we conducted a meta-analysis of longitudinal cohort studies to quantitatively evaluate the association between baseline TyG index and the risk of developing CKD in adults.

## Methods

This meta-analysis followed the PRISMA 2020 guidelines [28,29] and the Cochrane Handbook for Systematic Reviews and Meta-Analyses [28] for protocol design, data extraction, statistical analysis, and results reporting. The study protocol was also registered in PROSPERO under ID CRD420251030042.

### Literature search

Relevant studies for this meta-analysis were identified through a comprehensive search in PubMed, Embase, and Web of Science using a broad range of search terms, which included: (“TyG index” OR “triglyceride-glucose index” OR “triglyceride and glucose index” OR “triglyceride glucose index” OR “triacylglycerol glucose index” OR “TyGI”) AND (“chronic kidney disease” OR “CKD” OR “glomerular filtration rate” OR “renal function” OR “chronic renal failure”). The search was limited to human studies and full-length articles published in English or Chinese in peer-reviewed journals. Additionally, references from relevant original and review articles were manually screened for further eligible studies. The search spanned from database inception to March 05, 2025. The full search strategy for each database is shown in Supplemental File 1.

### Inclusion and exclusion criteria

The eligibility criteria for studies were established based on the PICOS framework:

P (patients): Adult populations (aged ≥18 years), either diabetic or nondiabetic, who were without CKD at baseline.
I (exposure): Participants with a high TyG index at baseline. The cutoffs for defining a high TyG index were consistent with the values used in the original studies.
C (comparison): Participants with a low TyG index at baseline.
O (outcome): Incidence of CKD during follow-up, compared between adults with a high versus a low TyG index at baseline. The minimal follow-up duration was one year. In general, CKD was defined as a decline in estimated glomerular filtration rate (eGFR) to ≤60 mL/min/1.73 m^2^ and/or the presence of albuminuria, based on standard clinical guidelines. Studies that assessed CKD incidence based on self-reported data were excluded, as self-reporting may lead to misclassification and introduce bias in outcome assessment due to recall inaccuracies and lack of clinical verification.
S (study design): Longitudinal observational studies, including cohort studies, nested case-control studies, or post-hoc analyses of clinical trials.

Studies were excluded if they were reviews, editorials, meta-analyses, preclinical research, cross-sectional studies, involving pediatric patients, lacked TyG index as the exposure, or did not report CKD incidence during follow-up. When population overlap occurred, the study with the largest sample size was selected for inclusion in the meta-analysis.

### Study quality assessment and data extraction

Two authors independently conducted the literature search, study selection, quality assessment, and data extraction, resolving discrepancies through discussion with the corresponding author. Study quality was evaluated using the Newcastle–Ottawa Scale (NOS) [30], which assesses selection, confounding control, and outcome measurement, with scores ranging from 1 to 9, where 9 represents the highest quality. Studies with NOS scores of 7 or above are considered of high quality. Data extracted for analysis included study characteristics (first author, publication year, study design, and country), participant information (baseline characteristics, sample size, mean age, sex distribution, diabetic status, and mean body mass index [BMI]), methods used to determine the TyG index cutoff, the baseline TyG index cutoff value, follow-up duration, CKD definition, number of participants who developed CKD during follow-up, and covariates adjusted for in the analysis of the association between TyG index and incident CKD. We carefully assessed the possibility of overlapping populations by comparing study settings, timeframes, cohorts, and author groups. No overlapping datasets were identified among the included studies.

### Statistical analyses

The association between TyG index at baseline and the incidence of CKD was expressed as risk ratios (RRs) with 95% confidence intervals (CIs), compared between adults with a high versus a low TyG index at baseline. Hazard ratios (HRs) from prospective cohort studies were directly treated as RRs, given their comparable interpretation when the outcome is relatively rare. Odds ratios (ORs) from retrospective studies were converted to RRs using a previously validated formula, to ensure consistency of effect estimates across studies. Briefly, RR = OR/{(1 − p_ref) + (p_ref × OR)}, where p_ref is the outcome risk in the reference (low-TyG) group [31]. RRs and their standard errors were calculated from 95% CIs or *p*-values and log-transformed to stabilize variance and normalize distribution [28]. To assess heterogeneity, we used the Cochrane *Q* test and *I*^2^ statistics [32], with *I*^2^ < 25% considered low, 25–50% moderate, 50–75% substantial, and >75% considerable heterogeneity among the included studies. Although statistical heterogeneity was low (*I*^2^ = 7%), a random-effects model was used to account for clinical and methodological variability across studies, including differences in population characteristics, TyG index cutoffs, and CKD definitions. This approach was considered more conservative and appropriate for observational data [28]. In addition, a sensitivity analysis using a fixed-effects model was also performed. Moreover, sensitivity analysis was also conducted by sequentially excluding individual studies to assess the robustness of the findings. Moreover, a sensitivity analysis limited to studies with prospective design was also performed to validate the stability of the finding. In addition, subgroup analyses were performed to explore the effects of various factors on the results, such as study population (general community-derived adult population versus population at higher risk for CKD, such as older population, people with diabetes, hypertension, or metabolic dysfunction-associated fatty liver disease [MAFLD] etc.), mean ages of the participants, proportions of men, mean BMI at baseline, methods for determining the cutoff of TyG index, cutoff values for defining a high TyG index at baseline, mean follow-up durations, and definitions of CKD. For studies that analyzed continuous variables, we used the reported median values as cutoff points in subgroup analyses. Publication bias was assessed through funnel plots, visual asymmetry inspection, and Egger’s regression test [33]. A *p* value < 0.05 indicates statistical significance. The statistical analyses were conducted using RevMan (Version 5.1; Cochrane Collaboration, Oxford, UK) and Stata software (version 12.0; Stata Corporation, College Station, TX, USA).

## Results

### Study identification

**Figure 1.**
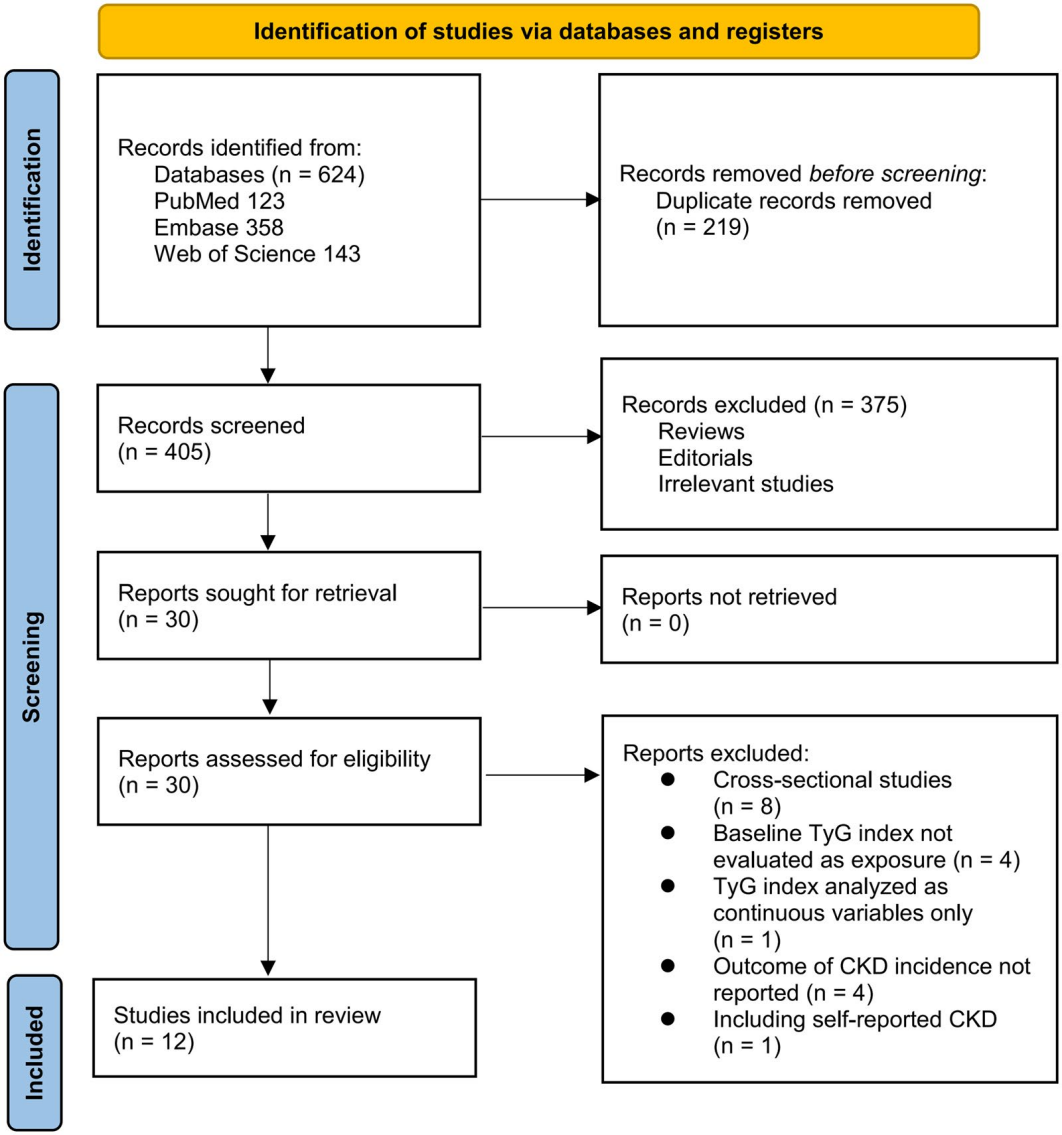
Flowchart of database search and study inclusion.

The study selection process is illustrated in Figure 1. A total of 624 records were retrieved from three databases, and 219 duplicates were removed. Following screening of titles and abstracts, 375 records were excluded based on predefined eligibility criteria. The full texts of 30 articles were then assessed in detail by two independent reviewers, resulting in the exclusion of 18 studies for reasons outlined in Figure 1. Ultimately, 12 cohort studies were deemed eligible and included in the meta-analysis [16–27].

### Overview of the study characteristics

Table 1 presents a summary of the characteristics of the studies included in the meta-analysis. Because one study reported the outcome in men and women respectively, these datasets were independently included in the meta-analysis [16]. Overall, seven prospective cohort studies [16–19,23,24,27] and five retrospective cohort studies [20–22,25,26] were involved in the meta-analysis. These studies published between 2019 and 2024, encompassing both prospective and retrospective designs. These studies were conducted across Japan, China, and Finland, with sample sizes ranging from 424 to 11,860 participants. Accordingly, 58,838 adults were included in the meta-analysis. The populations varied from general community residents [17,19,23,24,27] and individuals undergoing health checkups [16,26] to specific subgroups such as older people [20,21], patients with type 2 diabetes mellitus (T2DM) [18], MAFLD [25], and those with hypertension and abnormal glucose metabolism [22]. The mean age of participants ranged from approximately 40.9 to 70.8 years. The BMI averaged between 20.7 and 27.4 kg/m^2^. The included studies used quartiles [19–23,25–27], tertiles [17,18,24], or medians 16-based comparisons to define high TyG index levels, with the cutoff values of a high TyG index varying from 7.4 to 9.5. The follow-up durations ranged from 1.8 to 17.5 years, with a mean follow-up duration of 5.7 years. CKD was consistently defined using eGFR ≤60 mL/min/1.73 m^2^, and supplemented with proteinuria or urine albumin-to-­creatinine ratio thresholds in some of the included studies [17–19,22–26]. The number of CKD cases ranged from 94 to 2,005 across studies, and a total of 6,376 (10.8%) participants had newly developed CKD. Multivariate analyses were performed in all studies when the association between TyG index and the risk of CKD was reported, adjusting for key demographic and clinical covariates, such as age, sex, BMI, blood pressure, lipid profiles, glycemic parameters, and baseline kidney function to a varying degree. The NOS scores of the included studies ranged from seven to nine, indicating high methodological and reporting quality (Table 2).

**Table 1.** Characteristics of the included cohort studies.

Study	Design	Country	Participants	Sample size	Mean age (years)	Men (%)	DM (%)	Mean BMI (kg/m^2^)	Methods for determining cutoff of TyG index	Cutoff values for a high TyG index	Follow-up duration (years)	Definition of CKD	No. of subjects with CKD	Variables adjusted
Okamura 2019 men [16]	PC	Japan	Participants of healthcare examination	6026	41.1	100	0	22.9	Median	8.3	4	eGFR <60 mL/min/1.73 m^2^	120	Age, BMI, WC, smoking, exercise, alcohol consumption, SBP, albumin, HbA1c, hyperuricemia, HDL-C, LDL-C, CRP, SCr, and GGT
Okamura 2019 women [16]	PC	Japan	Participants of healthcare examination	5686	40.9	0	0	20.7	Median	7.7	3.7	eGFR <60 mL/min/1.73 m^2^	141	Age, BMI, WC, smoking, exercise, alcohol consumption, SBP, albumin, HbA1c, hyperuricemia, HDL-C, LDL-C, CRP, SCr, and GGT
Lv 2021 [18]	PC	China	T2DM patients	424	61	57.9	100	24.3	T3:T1	9.5	1.8	eGFR <60 mL/min/1.73 m^2^ or UACR >30 mg/g over 3 months	94	Age, sex, DM duration, BMI, hypertension, hypoglycemic therapy, hypolipidemic therapy, and anti-hypertension medications
Gao 2021 [17]	PC	China	Community population	2446	59.2	39.6	35.2	25.2	T3:T1	NR	3.9	eGFR ≤60 mL/min per 1.73 m^2^ or albuminuria	230	Age, sex, status of current smoking or drinking, education levels, physical activity, HbA1c, PP, HDL-C LDL-C, TC, BMI and use of ACEI/ARB
Xu 2021 [19]	PC	China	Community population	3439	56.8	30.2	9	NR	Q4:Q1	9.2	3.1	eGFR <60 mL/min/1.73 m^2^ or UACR ≥30 mg/g over 3 months	238	Age, sex, education, smoking, hypertension, DM, HbA1c, HDL-C, LDL-C, TC, 25 (OH)D3, BMI and TG/HDL-C
Zhu 2022 [22]	RC	China	Participants with hypertension and abnormal glucose metabolism	2033	55.5	56.5	58.1	27.4	Q4:Q1	9.4	2.6	eGFR <60 mL/min/1.73 m^2^ or positive proteinuria	302	Age, sex, smoking, alcohol drink, BMI, duration of hypertension, duration of DM, SBP, DBP, LDL-C, HDL-C, Cr, BUN, and UA, lipid-lowering drugs, antidiabetic drugs, and antihypertensive drugs
Lei 2022 [20]	RC	China	Older community people aged ≥65 years	7822	70.8	40.6	20	22.5	Q4:Q1	9.1	2	eGFR <60 mL/min/1.73 m^2^	1541	Age, sex, exercising daily, alcohol drinking daily, and current smoking status, DM, SBP, DBP, BMI, WC, baseline eGFR, and TC
Xiong 2022 [21]	RC	China	Older community people aged ≥65 years	2436	65	38.5	16	23.1	Q4:Q1	8.1	5	eGFR <60 mL/min/1.73 m^2^	273	Age, sex, BMI, hypertension, dyslipidemia, and SUA at baseline
Chen 2024 23	PC	China	Community population	5484	52.5	45.3	0	24.2	Q4:Q1	9	3.8	eGFR <60 mL/min/1.73 m^2^ or UACR >300 mg/g	879	Age, sex, current smoking, current alcohol drinking, educational level, physical activity, hypertension, BMI, baseline LDL-C, HDL-C, ALT, AST, SCr, UA, Urea, and baseline eGFR
Liu 2024 [27]	PC	China	Community people aged ≥45 years	3899	59	45.9	15.3	23.5	Q4:Q1	9	4	eGFR <60 mL/min/1.73 m^2^	191	Age, sex, SBP, DBP, TC, HbA1c and baseline eGFR, smoking, alcohol drinking, marriage, residence, education and medication usage
Hou 2024 [26]	RC	China	Participants of healthcare examination	4921	54.6	63.8	9.7	24	Q4:Q1	7.4	8	eGFR <60 mL/min/1.73 m^2^ or UACR >30 mg/g over 3 months	139	Age, sex, smoking status, alcohol consumption status, hypertension status, BMI, and SUA at baseline
Wei 2024 [25]	RC	China	Adults with MAFLD	11860	45.1	76.4	18.8	26.7	Q4:Q1	9.4	10	eGFR <60 mL/min/1.73 m^2^ or positive proteinuria	2005	Age, sex, hypertension status, DM, dyslipidemia status, and BMI, TC, LDL, AST, ALT, and baseline eGFR
Kunutsor 2024 [24]	PC	Finland	Community men aged 42 ∼ 61 years	2362	53	100	4	26.9	T3:T1	NR	17.5	eGFR <60 mL/min/1.73 m^2^ or positive proteinuria over 3 months	223	Age. BMI, SBP, TC, smoking, prevalent DM, hypertension and CAD, alcohol consumption, SES, eGFR at baseline, and physical activity

25(OH)D3, 25-hydroxyvitamin D3; ACEI, angiotensin-converting enzyme inhibitor; ALT, alanine aminotransferase; ARB, angiotensin receptor blocker; AST, aspartate aminotransferase; BMI, body mass index; BUN, blood urea nitrogen; CAD, coronary artery disease; CKD, chronic kidney disease; Cr, creatinine; CRP, C-reactive protein; DBP, diastolic blood pressure; DM, diabetes mellitus; eGFR, estimated glomerular filtration rate; GGT, gamma-glutamyl transferase; HbA1c, hemoglobin A1c; HDL-C, high-density lipoprotein cholesterol; LDL-C, low-density lipoprotein cholesterol; MAFLD, metabolic dysfunction-associated fatty liver disease; NR, not reported; PC, prospective cohort; PP, pulse pressure; RC, retrospective cohort; SBP, systolic blood pressure; SCr, serum creatinine; SES, socioeconomic status; SUA, serum uric acid; TC, total cholesterol; TG, triglyceride; TyG, triglyceride-glucose; UA, uric acid; UACR, urine albumin-to-creatinine ratio; WC, waist circumference.

**Table 2.** Study quality evaluation *via* the Newcastle-Ottawa scale.

Study	Representativeness of the exposed cohort	Selection of the non-exposed cohort	Ascertainment of exposure	Outcome not present at baseline	Control for age and sex	Control for other confounding factors	Assessment of outcome	Enough long follow-up duration	Adequacy of follow-up of cohort	Total
Okamura 2019 men [16]	1	1	1	1	1	1	1	1	1	9
Okamura 2019 women [16]	1	1	1	1	1	1	1	1	1	9
Lv 2021 [18]	1	1	1	1	1	1	1	0	1	8
Gao 2021 [17]	1	1	1	1	1	1	1	1	1	9
Xu 2021 [19]	1	1	1	1	1	1	1	1	1	9
Zhu 2022 [22]	0	1	1	1	1	1	1	0	1	7
Lei 2022 [20]	0	1	1	1	1	1	1	0	1	7
Xiong 2022 [21]	0	1	1	1	1	1	1	1	1	8
Chen 2024 [23]	1	1	1	1	1	1	1	1	1	9
Liu 2024 [27]	1	1	1	1	1	1	1	1	1	9
Hou 2024 [26]	0	1	1	1	1	1	1	1	1	8
Wei 2024 [25]	0	1	1	1	1	1	1	1	1	8
Kunutsor 2024 [24]	1	1	1	1	1	1	1	1	1	9

### TyG index and risk of CKD

**Figure 2.**
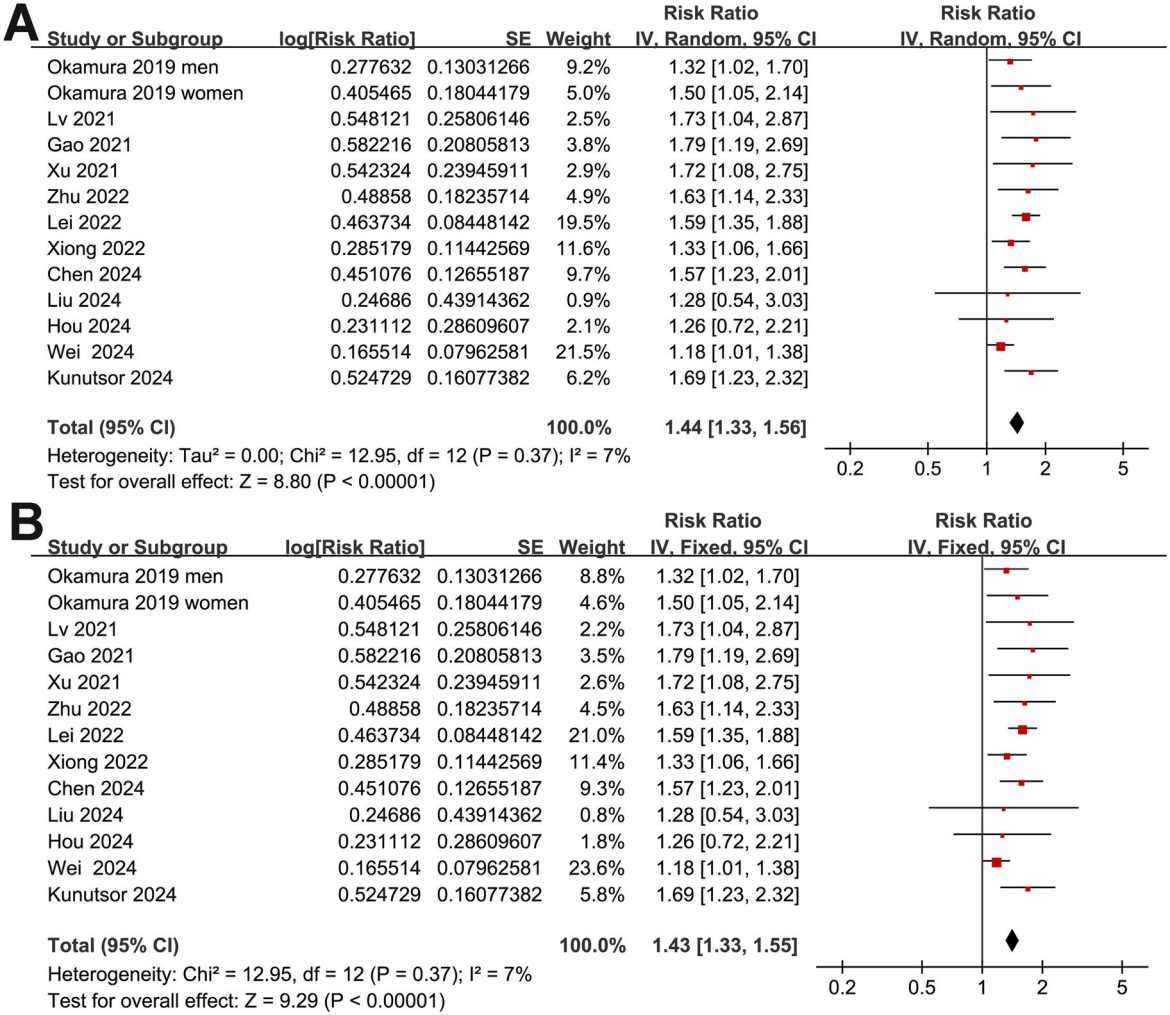
Forest plots for the meta-analysis of the association between TyG index and the incidence of CKD in adults; A, overall meta-analysis with a random-effects model; and B, sensitivity analysis using a fixed-effects model.

Overall, 13 datasets from 12 cohort studies [16–27] reported the association between baseline TyG index and the risk of CKD in adult populations, showing mild heterogeneity (Cochrane *Q* test *p* = 0.37; *I*^2^ = 7%). The pooled analysis using a random-effects model demonstrated that a higher TyG index at baseline was significantly associated with an increased risk of incident CKD during follow-up (RR: 1.44, 95% CI: 1.33–1.56, *p* < 0.001; Figure 2A). A sensitivity analysis using a fixed-effects model showed similar results (RR: 1.43, 95% CI: 1.33–1.55, *p* < 0.001; Figure 2B). Sensitivity analyses, performed by sequentially excluding one dataset at a time, revealed stable results (RR range: 1.39–1.52, all *p* < 0.05). A separate sensitivity analysis restricted to prospective cohort studies also yielded consistent findings (RR: 1.55, 95% CI: 1.37–1.75, *p* < 0.001), with no significant heterogeneity (Cochrane *Q* test *p* = 0.90; *I*^2^ = 0%).

Results of subgroup analyses are shown in Table 3. Subgroup analyses indicate that the positive association between TyG index and CKD risk remained consistent across different population subgroups, including general adults and those at higher risk for CKD (e.g. older adults, individuals with T2DM, hypertension, or MAFLD; RR: 1.52 vs. 1.41, *p* for subgroup difference = 0.47), participants with mean age <55 or ≥55 years (RR: 1.37 vs. 1.55, *p* = 0.17), studies with <50% or ≥50% male participants (RR: 1.53 vs. 1.38, *p* = 0.24), and those with mean BMI ≤24 or >24 kg/m^2^ (RR: 1.45 vs. 1.51, *p* = 0.69). Moreover, the association was not significantly influenced by the method used to determine TyG index cutoffs (*p* = 0.26) or the specific cutoff values applied to define high TyG index (*p* = 0.64). Notably, a stronger association was observed in studies with follow-up durations <4 years compared to those with longer follow-up (RR: 1.61 vs. 1.29, *p* for subgroup difference = 0.004). Finally, the association remained robust regardless of CKD definition—whether based on reduced eGFR alone or a combination of low eGFR and/or proteinuria (RR: 1.46 vs. 1.49, *p* = 0.79).

**Table 3.** Results of subgroup analyses.

Variables	No. of datasets	RR (95% CI)	*I* ^2^	*p* for subgroup effects	*p* for subgroup difference
Population					
General adults	8	1.52 [1.34, 1.72]	0%	<0.001	
Elderly/DM/HTN/MAFLD	5	1.41 [1.21, 1.65]	52%	<0.001	0.47
Mean age					
<55 years	6	1.37 [1.21, 1.56]	25%	<0.001	
≥55 years	7	1.55 [1.38, 1.73]	0%	<0.001	0.17
Men					
<75%	7	1.53 [1.38, 1.70]	0%	<0.001	
≥50%	6	1.38 [1.19, 1.59]	2%	<0.001	0.24
Mean BMI					
BMI ≤24 kg/m^2^	6	1.45 [1.30, 1.61]	0%	<0.001	
BMI >24 kg/m^2^	6	1.51 [1.27, 1.79]	48%	<0.001	0.69
Methods for TyG index cutoff					
Median	2	1.38 [1.12, 1.70]	0%	0.002	
T3:T1	3	1.73 [1.38, 2.16]	0%	<0.001	
Q4:Q1	8	1.42 [1.27, 1.59]	26%	<0.001	0.26
Cutoff values of TyG index					
≤9	6	1.40 [1.24, 1.59]	0%	<0.001	
>9	5	1.48 [1.23, 1.78]	55%	<0.001	0.64
Follow-up durations					
<4 years	7	1.61 [1.44, 1.79]	0%	<0.001	
≥4 years	6	1.29 [1.16, 1.43]	0%	<0.001	0.004
Definition of CKD					
eGFR <60 mL/min/1.73 m^2^	5	1.46 [1.30, 1.63]	0%	<0.001	
eGFR <60 mL/min/1.73 m^2^ or proteinuria	8	1.49 [1.29, 1.72]	33%	<0.001	0.79

RR, relative risk; CI, confidence interval; DM, diabetes mellitus; HTN, hypertension; MAFLD, metabolic dysfunction-associated fatty liver disease; BMI, body mass index; TyG, triglyceride-glucose; T3:T1, tertile 3 versus tertile 1; Q4:Q1, quartile 4 versus quartile 1; CKD, chronic kidney disease; eGFR, estimated glomerular filtration rate.

### Publication bias

**Figure 3.**
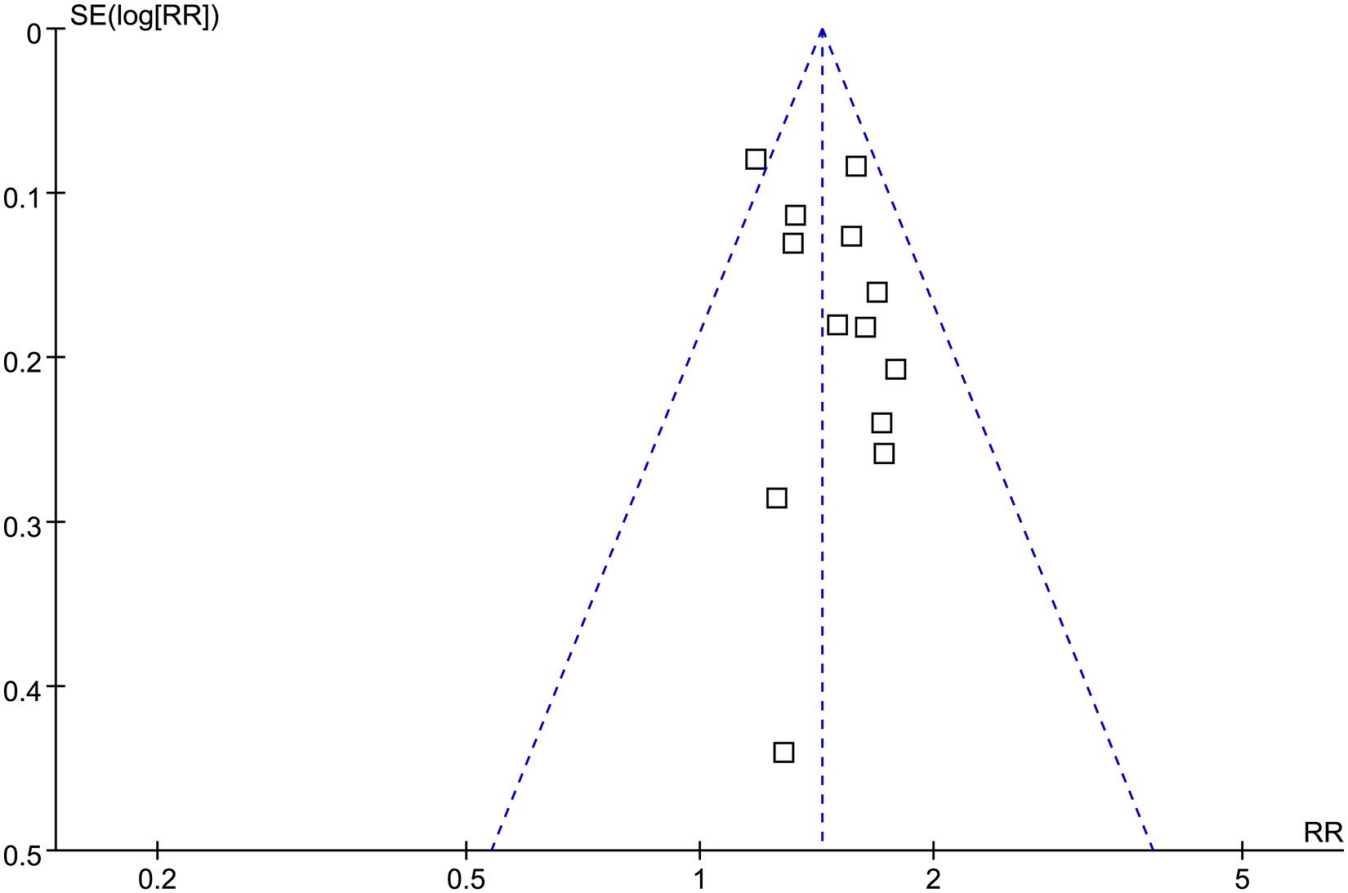
Funnel plots for estimating the potential publication bias underlying the meta-analysis of the association between TyG index and the incidence of CKD in adults.

Figure 3 displays funnel plots of the meta-analysis evaluating the association between TyG index and the risk of CKD. The plots were symmetrical on visual inspection, suggesting a low risk of publication bias. These findings are further supported by Egger’s regression analyses, which did not suggest a significant publication bias either (*p* = 0.72).

## Discussion

This meta-analysis of 13 datasets from 12 longitudinal cohort studies involving 58,838 adults demonstrated that a TyG index at baseline is significantly associated with an increased risk of incident CKD during follow-up. Sensitivity analyses, including leave-one-out analysis and restriction to prospective studies only, confirmed the robustness of the association. Subgroup analyses further revealed that the association remained consistent across various populations, age groups, sex distributions, BMI categories, and TyG index definitions. Notably, a stronger association was observed in studies with follow-up durations of less than four years compared to those with longer follow-up periods, indicating the predictive utility of TyG index may be more evident in short-term CKD risk assessment.

The observed association may be explained by several biological mechanisms linking insulin resistance, reflected by the TyG index, to the pathogenesis of CKD. Elevated fasting glucose levels contribute to renal injury through pathways including hyperglycemia-induced oxidative stress, inflammation, and glycation end-product accumulation, all of which damage glomerular structure and function [34,35]. Concurrently, elevated triglyceride levels are known to promote lipotoxicity, endothelial dysfunction, and mesangial cell injury, further aggravating renal impairment [36]. Clinically, the combination of elevated glucose and triglycerides, as captured by the TyG index, reflects a state of metabolic derangement that contributes to both glomerular hemodynamic changes and progressive nephron loss [10]. The TyG index may thus serve as an integrative marker that captures multiple metabolic insults affecting kidney health [10].

The subgroup analyses offered additional insights. The association between TyG index and CKD risk was not significantly modified by population characteristics, including age, sex, BMI, or baseline risk profile, suggesting that the TyG index is broadly applicable across demographic and clinical subgroups. However, the stronger association observed in studies with shorter follow-up durations (<4 years) raises important questions. One possible explanation is that individuals with a high TyG index may already have subclinical renal impairment at baseline, which manifests as clinically detectable CKD within a few years [37,38]. Alternatively, the impact of metabolic disturbances may be most pronounced in the early stages of renal decline, whereas in longer follow-up periods, other competing factors (such as aging or comorbidities) may dilute the effect of the TyG index. Importantly, this pattern also raises the possibility of reverse causation, whereby undetected early kidney dysfunction could contribute to both elevated TyG levels and subsequent CKD diagnosis. Therefore, the subgroup finding should be interpreted with caution. This finding underscores the importance of early metabolic screening and intervention.

This study has several strengths. All included studies were longitudinal cohorts, allowing for the temporal relationship between exposure and outcome to be assessed. The results were consistent across multiple sensitivity and subgroup analyses, enhancing the credibility of the findings. Furthermore, all studies adjusted for key demographic and clinical variables in multivariate models, reducing the likelihood of confounding. The large combined sample size and low heterogeneity further strengthen the reliability and generalizability of the results. Nonetheless, several limitations should be acknowledged. First, there was variability in the baseline characteristics of study populations, including age, prevalence of comorbidities, and the risk profile for CKD, which may have influenced the observed associations. Second, the included cohorts applied slightly different criteria to define CKD, with some relying solely on reduced eGFR and others combining eGFR with albuminuria or proteinuria, which may have introduced variability in outcome classification. Third, the TyG index cutoff values used to define “high” TyG varied widely across studies (range: 7.4–9.5) and were based on different statistical methods (e.g. quartiles, tertiles, or medians). Two studies [17,24] did not report numerical thresholds but applied internal percentile-based classifications. Moreover, our analyses were based on categorical comparisons of high versus low TyG index, as the majority of studies reported results in this format; thus, a dose–response relationship (per unit or per SD increase) could not be assessed. While this heterogeneity limits the direct clinical translation of an optimal TyG threshold, our subgroup analyses demonstrated that the association between elevated TyG and CKD risk remained robust across different cutoff methods and values. This highlights the consistency of the signal across diverse populations and study designs. Nonetheless, future research is warranted to establish standardized and clinically validated TyG index thresholds for effective risk stratification. Additionally, although multivariate adjustments were made, residual confounding from unmeasured factors such as dietary intake [39], physical activity [40], and concurrent medication use such as statins [41] and sodium-glucose co-transporter 2 inhibitors [42] cannot be ruled out. These variables were either inconsistently reported or not included in most multivariate models, which may limit the ability to fully isolate the independent effect of TyG index on CKD risk. In addition, all included studies, used only a single baseline measurement of TyG index, which may not reflect longitudinal changes in metabolic status or capture cumulative exposure over time. This static exposure assessment may underestimate or obscure the true relationship between TyG dynamics and CKD development. Future studies incorporating repeated measures or trajectory analyses are warranted. Finally, as this is an observational meta-analysis, causality cannot be established.

From a clinical perspective, the findings support the potential utility of the TyG index as a simple, inexpensive, and accessible tool for identifying individuals at increased risk of CKD. Given its reliance on routinely available laboratory parameters, the TyG index could be easily incorporated into primary care settings to aid in early risk stratification and prompt preventive strategies [43]. This is particularly valuable in resource-limited settings where more sophisticated assessments of insulin resistance are not feasible [43]. However, before clinical implementation, consensus on standardized cutoff values and further validation across diverse populations are needed. Future research should focus on prospective interventional studies to evaluate whether lowering the TyG index through lifestyle or pharmacological approaches can reduce CKD risk. Moreover, longitudinal studies examining changes in TyG index over time in relation to kidney function trajectories would provide deeper insights into its dynamic relationship with renal health. The role of TyG index in predicting CKD progression and other renal outcomes, such as end-stage kidney disease or albuminuria, also warrants further investigation.

## Conclusions

In conclusion, this meta-analysis provides robust evidence that an elevated TyG index at baseline is associated with an increased risk of incident CKD in adults. The association appears consistent across a range of populations and clinical characteristics and is particularly pronounced in the short term. These findings highlight the TyG index as a promising marker for early detection of individuals at risk of kidney function decline, supporting its potential application in preventive nephrology and metabolic risk management.

## Supplementary Material

Supplemental File 1.docx
