# Supplementary material for: Association between triglyceride-glucose index and risk of chronic kidney disease: a meta-analysis
Source: Ren Fail. 2025 Oct 29;47(1):2572356. doi: 10.1080/0886022X.2025.2572356 (PMC12573563; doi:10.1080/0886022X.2025.2572356)
Supplement: Supplemental File 1.docx [file IRNF_A_2572356_SM9369.docx]

**Search strategy for each database**

**PubMed**

("TyG index"[All Fields] OR "triglyceride-glucose index"[All Fields] OR "triglyceride and glucose index"[All Fields] OR "triglyceride glucose index"[All Fields] OR "triacylglycerol glucose index"[All Fields] OR "TyGI"[All Fields])

AND

("chronic kidney disease"[MeSH Terms] OR "chronic kidney disease"[All Fields] OR "CKD"[All Fields] OR "chronic renal failure"[MeSH Terms] OR "chronic renal failure"[All Fields] OR "glomerular filtration rate"[MeSH Terms] OR "glomerular filtration rate"[All Fields] OR "renal function"[All Fields])

AND

("diabetes mellitus"[MeSH Terms] OR "diabetes"[All Fields] OR "diabetic"[All Fields])

**Embase**

('tyg index':ti,ab OR 'triglyceride-glucose index':ti,ab OR 'triglyceride and glucose index':ti,ab OR 'triglyceride glucose index':ti,ab OR 'triacylglycerol glucose index':ti,ab OR 'tygi':ti,ab)

AND

('chronic kidney disease'/exp OR 'chronic kidney disease':ti,ab OR 'ckd':ti,ab OR 'chronic renal failure'/exp OR 'chronic renal failure':ti,ab OR 'glomerular filtration rate'/exp OR 'glomerular filtration rate':ti,ab OR 'renal function':ti,ab)

AND

('diabetes mellitus'/exp OR 'diabetes':ti,ab OR 'diabetic':ti,ab)

**Web of Science**

TS=("TyG index" OR "triglyceride-glucose index" OR "triglyceride and glucose index" OR "triglyceride glucose index" OR "triacylglycerol glucose index" OR "TyGI")

AND

TS=("chronic kidney disease" OR "CKD" OR "chronic renal failure" OR "glomerular filtration rate" OR "renal function")

AND

TS=("diabetes" OR "diabetic")
